# Parvovirus B19 Infection Mimicking Juvenile Myelomonocytic Leukemia in an Immunocompetent Child

**DOI:** 10.7759/cureus.8854

**Published:** 2020-06-26

**Authors:** Aji Mathew, Walid Abuhammour, Basil Fathalla, Bosaina Otour

**Affiliations:** 1 Pediatric Pulmonology, Al Jalila Children Hospital, Dubai, ARE; 2 Infectious Diseases, Al Jalila Children's Speciality Hospital, Dubai, ARE; 3 Rheumatology, Al Jalila Children's Speciality Hospital, Dubai, ARE; 4 Paediatrics, Al Jalila Children's Speciality Hospital, Dubai, ARE

**Keywords:** parvovirus b19, juvenile myelomonocytic leukemia

## Abstract

Most of the parvovirus infections in humans are benign. Clinical manifestations of parvovirus B19 infections in children vary from erythema infectiosum in healthy children to aplastic crisis in patients with hematological disorders (such as sickle cell disease) and immunocompromised patients. Parvovirus B19 infects the erythroid progenitor cell in the bone marrow and causes transient erythroblastopenia. Transient leukoerythroblastic reaction is a rare presentation of parvovirus infection. Our case is a child presenting with fever of unknown origin (FUO) who was investigated and treated in different hospitals for FUO. He was investigated for infections, rheumatological causes, and malignancies. Clinical manifestations and bone marrow findings were mimicking juvenile myelomonocytic leukemia (JMML) but eventually diagnosed to have a parvovirus B19 infection, which resolved spontaneously in due course.

## Introduction

Human parvovirus B19 is a single-stranded DNA virus that belongs to the Parvoviridae family. Clinical manifestations of parvovirus B19 infections in children vary from erythema infectiosum in healthy children to aplastic crisis in patients with hematological disorders (such as sickle cell disease) and immunocompromised patients [1]. Parvovirus B19 infects the erythroid progenitor cell in the bone marrow and causes transient erythroblastopenia. It has been associated with transient red cell aplasia, aplastic crisis, and lupus like syndromes in children with hematological disorders and immunodeficiency [2,3]. Transient leukoerythroblastic reaction is a rare presentation of parvovirus infection [3,4].

We report herein a child presenting with fever of unknown origin (FUO) with clinical manifestations and bone marrow findings mimicking juvenile myelomonocytic leukemia (JMML) and leukoerythroblastic reaction who eventually diagnosed to have a parvovirus B19 infection.

## Case presentation

A previously healthy four-year-old Arab boy presented with a history of prolonged fever, generalized fatigue, and arthralgia of one-month duration. He was evaluated in other institutions without a conclusive diagnosis. He was treated with several courses of intravenous antibiotics without clinical response. Investigations during that phase of his illness revealed low hemoglobin of 9 gm/dl (normal range: 11-14) and raised inflammatory markers. On physical examination upon arrival to our center, he was noted to be febrile, pale, and ill looking. He had bilateral small 1 x 1 cm firm cervical lymph nodes, hepatosplenomegaly, and was unable to walk due to arthralgia of knees and ankles. Repeated workup after a week revealed a total while blood count (WBC) of 13.5 × 10^3^/mcl, an increased absolute neutrophil count (ANC) of 8.84 × 10^3^/mcl, a low absolute lymphocyte count (ALC) of 1.59 x 10^3^/mcl, a high absolute monocyte count (AMC) of 1.9 × 10^3^/mcl (0.2-1), a platelet count of 147,000/mcl, and a drop in his hemoglobin to 7.30 gm/dl (11-14). During his hospital course, he had persistent fever and the hemoglobin dropped further to 5.9 gm/dl after five days requiring packed red blood cell transfusion. Inflammatory markers were high including C-reactive protein (CRP) at 65 mg/dl (0-5), erythrocyte sedimentation rate (ESR) of 63 mm/hr (0-10), and ferritin of 145.5 mcg/L (4-60). Iron profile showed low serum iron and hemoglobin electrophoresis showed slightly elevated hemoglobin A2, which was suggestive of beta thalassemia trait. Direct Coombs test was negative, and G6PD screening was normal. Reticulocyte count was 0.59% (0.5%-2.5%) inappropriately low for the degree of anemia. Cultures of blood and urine were negative, and stool studies were unremarkable. Serological tests (IgG and IgM) and PCR for Epstein-Barr virus (EBV), Cytomegalovirus (CMV), Mycoplasma pneumoniae, Brucella species IgM and IgG, and enterovirus PCR were negative. Cerebrospinal fluid (CSF) analysis, culture, and virology studies, including Herpesvirus, CMV, and enterovirus, were normal. Screening for tuberculosis, including Quantiferon test and Mantoux skin test, was negative. Liver functions were normal except for a slightly low albumin of 3 g/dl (3.8-4.5). Antinuclear antibodies (ANA), anti-double stranded DNA, extractable nuclear antibodies (ENA) tests, rheumatoid factor tests, and antineutrophil cytoplasmic antibody (ANCA) tests (c-ANCA, p-ANCA, and atypical p-ANCA) were negative. He had normal immunoglobulin levels, including IgG and IgG subclasses, IgA, IgM, and IgE levels, and flow cytometry for T and B lymphocyte, and natural killer cells (NK) were normal. Tetanus, diphtheria, and pneumococcal antibody titers were normal. Serum triglycerides was 426 mmol/L (0-180) and soluble IL-2 receptor levels were high at 3,715 U/mL (223-710). New generation sequencing genetic testing for primary hemophagocytic lymphohistiocytosis panel was negative.

**Figure 1:**
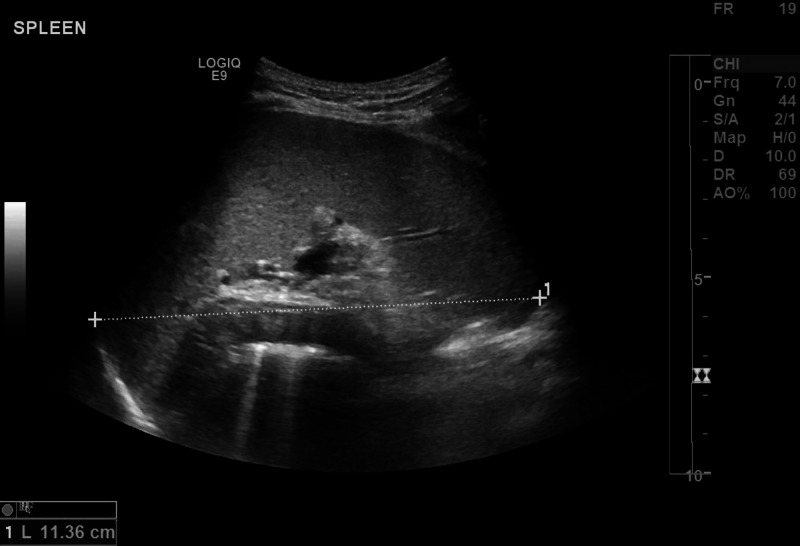
Mild splenomegaly at initial presentation

The blood film revealed severe microcytic hypochromic anemia with anisopoikilocytosis and eythrocytosis consistent with iron deficiency anemia combined with beta-thalassemia trait. WBC lineage showed leukoerythroblastic blood picture with leukocytosis, monocytosis, and subtle dysplastic neutrophils and was suggestive of JMML. In addition, radiological findings of abdomen ultrasonography (Figure 1) and CT of abdomen and pelvis showed mild hepatosplenomegaly with mild ascites. Therefore, a bone marrow study was performed to rule out leukemia, which showed megaloblastic changes, mild dyserythropoiesis, and subtle dysgranulopoietic changes. The bone marrow was dysplastic and suspicious of JMML; however, cytogenetics analysis for JMML was negative. Fluorescent in situ hybridization (FISH study for t(9;22) (q34;q11.2) (BCR/ABL) DF, D7S486(7q31)/CEP 7, EGR1(5q31)/ D5S23,D5S721(5p15.2), and CEP 8) showed no chromosomal rearrangement.

**Figure 2:**
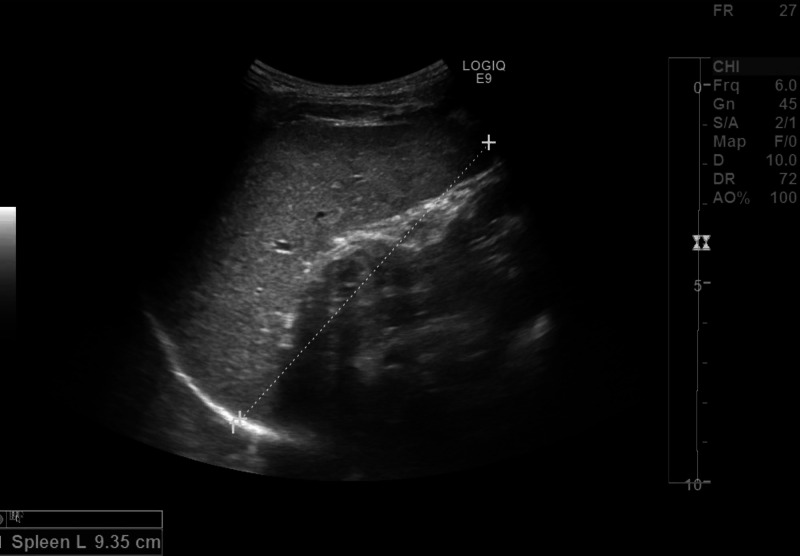
Regression of spenomegaly after three months of initial presentation

We pursued further investigations in view of lack of diagnosis. Although late in the course, we sent for parvovirus B19 blood PCR (qualitative) that detected presence of parvovirus B19 DNA. Serum parvovirus IgG titer was positive at 6.7 index units (positive >1.1) but IgM titer was negative. Meanwhile, fever started to subside gradually and he had gradual symptomatic improvement over the three-week period. His clinical examination upon discharge revealed resolution of hepatosplenomegaly (Figure 2), confirmed by repeated abdominal ultrasound. Repeated parvovirus B19 PCR after three months (this was done quantitative) was positive, and ultimately the PCR test became negative at six months after initial testing. The serum parvovirus IgG titer declined gradually to 5 index units (positive >1.1) in two months and then 3.6 index units (positive >1.1) at six months. Further follow-up during 12 months revealed the child to be doing well with no symptoms and normal physical examination. 

## Discussion

Majority of parvovirus B19 infections are benign and asymptomatic in healthy children. The most common presentation is erythema infectiosum which is a self-limiting disorder. However, parvovirus infection can be life threatening in specific populations. Bone marrow suppression and red cell aplasia may develop in mostly patients who are immunocompromised or with hematological disorders like sickle cell disease and other hemoglobinopathies, and rarely in healthy children [5,6]. There are few reports of parvovirus B19 infections presenting with a prolonged course and unexpected complications, such as myocarditis and meningoencephalitis [7,8]. Rarely, parvovirus B19 infection can trigger a clinical course similar to systemic lupus erythematosus with nephritis, arthritis, vasculitis, and nephrotic syndrome [9]. Similar to other infections, parvovirus B19 infection can occasionally trigger transient autoantibody response, including ANA and rheumatoid factor.

Leukoerythroblastosis is a rare phenomenon that is characterized by leukocytosis and the presence of erythroid and myeloid blasts in the peripheral blood. In children, this blood picture is rare and is seen in JMML, myelofibrosis, metastatic bone marrow disease, and sometimes following viral infections. Two cases of neonatal parvovirus B19 infection presenting as transient myeloproliferation resembling JMML have been reported [10,11]. There is another reported case of an 11-month-old immune competent child presented as JMML that was also proved to be due to parvovirus B19 infection [12]. Myelodysplastic features mimicking JMML have been described in other viral diseases, such as acquired Immunodeficiency syndrome, HHV6, CMV, and EBV infection [13,14].

The present case was initially suspected to be JMML in view of the clinical features of hepatosplenomegaly with anemia and dysplastic bone marrow findings. Parvovirus B19 infection as a cause of the clinical and laboratory manifestations in this case was not initially suspected, and serology as well as PCR studies were not done as part of the initial workup. However, when cytogenetic studies for JMML were negative, further workup to identify an etiology was done, including workup for parvovirus B19. Although the parvovirus B19 IgM titer was negative, we believe that the etiology was parvovirus B19 infection. This child had complete resolution of hepatosplenomegaly, anemia, and lymphadenopathy, and normalization of laboratory markers without definitive treatment. The lack of alternative etiology, positive IgG titers that declined over time, positive parvovirus PCR that eventually became negative, and spontaneous resolution of clinical features were supportive of the diagnosis of parvovirus B19 infection. In selected cases of parvovirus infection with persistent anemia, intravenous immunoglobulin (IVIG) can be a potential therapeutic option.

## Conclusions

Although majority of parvovirus infections are benign, it can have rare and serious presentations, including FUO, myocarditis, and malignancies. Therefore, parvovirus B19 should be considered and early workup, including serology and PCR tests, should be done in children presenting with FUO and anemia with poor bone marrow reactivity, even in the presence of other misleading clues like organomegaly.
